# Genome-wide identification of the *OMT* gene family in *Cucumis melo* L. and expression analysis under abiotic and biotic stress

**DOI:** 10.7717/peerj.16483

**Published:** 2023-12-14

**Authors:** Shuoshuo Wang, Chuang Wang, Futang Lv, Pengfei Chu, Han Jin

**Affiliations:** 1 Liaocheng University, Liaocheng, China; 2 Liaocheng Vocational & Technical College, Liaocheng, China

**Keywords:** Melon, *CmOMT* genes, Phylogenetic analysis, Expression analysis, Stress

## Abstract

**Background:**

O-methyltransferase (OMT)-mediated O-methylation is a frequent modification that occurs during natural product biosynthesis, and it increases the diversity and stability of secondary metabolites. However, detailed genome-wide identification and expression analyses of *OMT* gene family members have not been performed in melons. In this study, we aimed to perform the genome-wide identification of *OMT* gene family members in melon to identify and clarify their actions during stress.

**Methods:**

Genome-wide identification of *OMT* gene family members was performed using data from the melon genome database. The *Cucumis melo* OMT genes (*CmOMTs)* were then compared with the genes from two representative monocotyledons and three representative dicotyledons. The basic information, *cis*-regulatory elements in the promoter, predicted 3-D-structures, and GO enrichment results of the 21 *CmOMTs* were analyzed.

**Results:**

In our study, 21 *CmOMTs* (named *CmOMT1-21*) were obtained by analyzing the melon genome. These genes were located on six chromosomes and divided into three groups composed of nine, six, and six *CmOMTs* based on phylogenetic analysis. Gene structure and motif descriptions were similar within the same classes. Each *CmOMT* gene contains at least one *cis*-acting element associated with hormone transport regulation. Analysis of *cis*-acting elements illustrated the potential role of *CmOMTs* in developmental regulation and adaptations to various abiotic and biotic stresses. The RNA-seq and quantitative real-time PCR (qRT-PCR) results indicated that NaCl stress significantly induced *CmOMT6*/*9*/*14*/*18* and chilling and high temperature and humidity (HTH) stresses significantly upregulated *CmOMT14*/*18*. Furthermore, the expression pattern of *CmOMT18* may be associated with *Fusarium oxysporum* f. sp. *melonis* race 1.2 (FOM1.2) and powdery mildew resistance. Our study tentatively explored the biological functions of *CmOMT* genes in various stress regulation pathways and provided a conceptual basis for further detailed studies of the molecular mechanisms.

## Introduction

Melon (*Cucumis melo* L., 2n = 24) is an economically important and widely cultivated crop, and it ranks ninth in terms of global production among horticultural crops (Kong et al., 2016). It is a sweet, musky, fleshy fruit rich in vitamins, minerals, and health-promoting antioxidants. More than 32 million tons of melon were produced worldwide in 2017 (Zhao et al., 2019). However, various stressors, such as disease, pest, drought, and salt stress, have caused steep declines in melon yield and quality during growth (Wang et al., 2022). Therefore, melons have evolved complex defense mechanisms to manage such stress (Jander & Clay, 2011). In general, plants protect themselves from adverse stresses in numerous ways, including programmed cell death (PCD), antimicrobial substance secretion, and secondary metabolite and endogenous hormone production (Jing et al., 2017; Kud et al., 2019). Plant hormones, such as abscisic acid, salicylic acid, ethylene, and melatonin, play essential roles in response to abiotic and biotic stresses (Li & Li, 2019; Zeng et al., 2022). Nevertheless, plant genomes have evolved to adapt to environmental changes (Kaessmann, 2010). The main factors underlying gene family evolution are gene duplication and loss events, and most duplications are generated *via* whole-genome duplication (WGD) and small-scale duplication events (Panchy, Lehti-Shiu & Shiu, 2016; Elisabeth, Leng & Alexander, 2018). Under abiotic and biotic stress conditions, members of various gene families execute specific biological functions that interconnect to form networks that control plant resistance (Glover, Redestig & Dessimoz, 2016; Wang et al., 2022) and govern a wide range of plant biochemical pathways (Glover, Redestig & Dessimoz, 2016). Investigating the gene families implicated in resistance mechanisms is crucial for exploiting biotechnological tools to improve desirable agronomic traits, such as crop growth and productivity. Therefore, the current study was performed to identify and clarify the actions of gene families in melon.

O-methyltransferases (OMTs) constitute one of the three major plant methyltransferases, and they transfer the methyl group of S-adenosyl-L-methionine (SAM) to the hydroxyl group of several natural compounds, thus forming methyl ether derivatives (Roje, 2006; Struck et al., 2012). Plant *OMT* genes can be divided into two main classes based on their molecular weight and divalent ion dependence: caffeic acid OMT (COMT) and caffeoyl coenzyme a OMT (CCoAOMT) (Liu et al., 2015). CCoAOMT and COMT participate in lignin biosynthesis. Unlike CCoAOMT, which acts on caffeinated CoA and 5-hydroxyferulic acid CoA, COMT can act on a range of substrates, such as chalcones, caffeic acid, 5-hydroxyferulic acid, and 5-hydroxyferuloylester (Roje, 2006; Liu et al., 2015). OMT coordinates the methylation of some secondary metabolites. Based on sequence homology and substrate differentiation, the OMT gene family can be divided into three categories that mediate the methylation of flavonoids, caffeoyl-CoA, and small phenolic compounds (Noel et al., 2003). *FAOMT* and *VvAOMT* were verified to catalyze anthocyanin methylation in red grapevines and grapevines, respectively (Hugueney et al., 2009; Lücker, Martens & Lund, 2010). Zhang et al. (2021a); Zhang et al. (2021b) demonstrated that *AOMTs* are primarily involved in new functionalization or non-functionalization after tandem duplication events. Many functional natural compounds, such as flavonoids, alkaloids, plant antitoxins, and lignin precursors, are generated through enzyme-catalyzed modifications in response to biotic and abiotic stresses (Pichersky & Lewinsohn, 2011; Gana et al., 2013).

Because *OMT* genes have essential roles in plant secondary metabolism, their functions have been extensively studied in numerous plant species (Sun et al., 2019; Chang et al., 2021). Several studies have shown that ASMT acts as an end enzyme and limits plant melatonin biosynthesis (Zhang et al., 2014). In the last two steps of melatonin biosynthesis, ASMT (N-acetylserotonin *O*-methyltransferase) methylates NAS (*N*-acetylserotonin) into melatonin in the cytoplasm (Byeon et al., 2014). The *ASMT1* gene was cloned from *Oryza sativa* in 2015 and named *OsASMT1*, and studies have shown that *OsASMT2/3* is upregulated in healing tissues in *OsASMT2/3*-overexpression transgenic rice plants (Park, Byeon & Back, 2013). Currently, *ASMT* genes have been cloned from a variety of dicotyledonous plant species, such as *Arabidopsis* and *Hypericum perforatum* (Byeon et al., 2016; Zhou et al., 2021). Melatonin (n-acetyl-5-methoxytryptamine) is an essential bioactive molecule commonly found in animals and plants (Hardeland, Cardinali & Srinivasan, 2011; Arnao, 2014). Numerous studies have shown that melatonin plays an important regulatory role in plant growth and development and responses to biotic and abiotic stresses, such as saline-alkali stress, chilling stress, and powdery mildew infection (Sun et al., 2019; Chang et al., 2021; Zeng et al., 2022). Melatonin improves salinity tolerance in tomato through the systematic regulation of ionic, water, acid–base, and redox balance (Yan et al., 2019). At the initial stage of pathogen infection, exogenous melatonin can increase intracellular H_2_O_2_ content, and it acts upstream of salicylic acid (SA) and positively regulates its accumulation to govern plant disease resistance (Zeng et al., 2022). COMT, a multifunctional enzyme also reported to have ASMT activity, is a member of the OMT family that can methylate numerous secondary metabolites, such as phenylpropanoids, flavonoids, and alkaloids (Lam et al., 2007; Byeon et al., 2014). In addition to determining the roles of *COMT* genes in the model crops *Oryza sativa*, *Arabidopsis*, and *Solanum lycopersicum*, their functions in melatonin biosynthesis in other plants must be further characterized to understand their novel functions (Byeon et al., 2014; Byeon et al., 2015; Liu et al., 2019). Additionally, *COMT* genes with ASMT functions can be applied to improve crop yield and quality through genetic manipulation and rational breeding.

The OMT gene family has been reported to be directly or indirectly involved in abiotic and biotic stress tolerance, and the specific functions of some OMTs have been identified in a variety of crops. In *Solanum lycopersicum*, overexpression of *SlCOMT1* significantly increases melatonin accumulation and improves salt and drought tolerance (Sun et al., 2019). In *Oryza sativa*, *OsCOMT3* plays an important role in enhancing nematode resistance (Petitot et al., 2017). A total of 58 OMT genes were identified in *Citrus*, 27 of which were involved in the methylation of flavonoids and contributed to the functional study of CitOMT genes. Phylogenetic analysis of OMT genes from three representative plants revealed that these proteins were classified into the COMT and CCoAOMT subfamilies (Liu et al., 2015). In soybean, 55 *COMT* genes were divided into two groups, and *GmCOMT* genes were differentially expressed under various abiotic stresses, such as salt and drought stress (Zhang et al., 2021a; Zhang et al., 2021b). In watermelon, a total of 16 *ClOMT* genes were identified and classified into three groups, and *ClCOMT1* was significantly upregulated under multiple stress conditions (cold, drought, and salt stress), whereas in transgenic *Arabidopsis, ClCOMT1* overexpression enhanced abiotic stress resistance (Chang et al., 2021). However, few studies have focused on the involvement of this gene family and its members in abiotic and biotic stresses and their regulation in melons.

In this study, we aimed to perform the genome-wide identification of *OMT* gene family members in melon to identify and clarify their actions during stress using data from the melon genome database. The objectives of this study were to (i) identify and analyze *CmOMT* genes, (ii) analyze the expression profile of *CmOMT* genes in melon tissues, (iii) analyze the response of *CmOMT* genes to abiotic and biotic stresses, (iv) elucidate the evolution the *OMT* gene family, and (v) provide a basis for the screening of melon stress-related *CmOMT* candidate genes and their regulation. We identified the phylogenetic relationships of *CmOMT* genes with their homologs from other plants as well as their chromosome location, gene structure, and amino acid composition. Thereafter, we performed RNA-seq and quantitative real-time reverse transcription-polymerase chain reaction (qRT-PCR) assays of *CmOMT* genes to determine their tissue-specific expression and responses to abiotic and biotic stresses.

## Materials & Methods

### Plant and material

‘Yangjiaomi’ is widely cultivated in China and has become an important commercial cultivar because of its crunchy texture, juiciness, and sweet taste. ‘Yangjiaomi’ was growing in the plant culture room of Liaocheng University, Liaocheng, China. Melon seeds were germinated in an incubator at 28 °C and then sown in 50-hole cavity trays (grass: vermiculite: perlite = 1:1:1, v/v). After the seedlings had grown three leaves and heart-shaped leaves, the treatments were applied in a plant culture room (26 °C/20 °C, day/night) with a 16 h photoperiod. The same melon plants were selected for the abiotic stress treatments, which were set according to a previous study: NaCl stress (300 mM for 1, 3, 5 and 7 d), chilling stress (6 °C for 6 and 12 h), and high temperature and humidity (HTH) stress (day/night temperature at 45 °C/35 °C, soil moisture at 100%, and humidity at 90%) (Wang et al., 2016; Diao et al., 2020; Weng et al., 2022). The control and treated melons were collected to determine the relative expression of the *CmOMT* gene family. Each stress treatment group included 15 melon plants, and three biological replicates were performed for all treatment groups.

### Identification of O-methyltransferase (OMT) genes in *Cucumis melo* L.

The melon (DHL92) protein database (Melon protein v4.0) was downloaded from the Cucurbitaceae Genome Database (CuGenDB; http://cucurbitgenomics.org/). To comprehensively identify the melon *OMT* gene family members, we searched the NCBI (Conserved Domain Search, http://www.ncbi.nlm.nih.gov/Structure/cdd/wrpsb.cgi/) and PFAM websites (http://pfam.xfam.org/) and analyzed the conserved motifs and protein functions of the reported ASMT and COMT proteins. PF00891 was employed as the reference sequence for the Hidden Markov Model (HMM) analysis of the melon protein database, and proteins with an *e*-value ≤ 1 × *e* ^−10^ were selected as candidate OMT proteins (Liu et al., 2015; Chang et al., 2021). The candidate proteins were subjected to protein sequence identification using the SMART website (http://smart.embl-heidelberg.de/) (Letunic, Khedkar & Bork, 2017). Sequences without OMT structural domains or incomplete structural domains were removed, and the longest sequence was retained if redundant sequences were identified.

### Physicochemical properties and chromosomal mapping of *CmOMT* genes in *Cucumis melo* L.

The molecular weight, isoelectric point (PI), formula, instability index, aliphatic index, and grand average hydropathicity (GRAVY) of the *CmOMT* genes were analyzed using the online tool ExPASy (http://web.expasy.org/protparam/) with the default parameters. The melon genome website (http://cucurbitgenomics.org/organism/18) provides the position information of *CmOMT* genes using MapChart software to map the distribution of *CmOMT* genes on chromosomes. The identification criteria for tandem duplication genes were as follows: (i) adjacent homologous genes were located on the same chromosome, with only one intercalated gene in the middle, and (ii) the comparison length and similarity of the two gene sequences were more than 70% (Zhu et al., 2014; Zhao et al., 2018).

### Phylogenetic analysis, exon-intron structure, and motif analysis

Multiple alignments of the amino acid sequences of the 21 CmOMT proteins were performed using the ClustalW tool with default parameters (Larkin et al., 2007). To investigate the phylogenetic relationships of OMT homolog protein sequences among six species (*Arabidopsis thaliana*, *Cucumis melo* L., *Cucumis sativus* L., *Solanum lycopersicum* L., *Oryza sativa* L., and *Citrullus lanatus* (Thunb.) Matsum. et Nakai), a phylogenetic tree was constructed using the neighbor-joining algorithm of MEGA X software. A phylogenetic tree was constructed using the neighbor-joining Jones Taylor–Thornton (JTT) matrix model in MEGA X software, where bootstrap repetitions were set to 1,000 to assess the plausibility of the evolutionary tree (Kumar, Stecher & Tamura, 2016). The phylogenetic tree was visualized using Figtree v1.4.3 (http://tree.bio.ed.ac.uk/software/figtree/).

The *CmOMT* gene structure was analyzed using the Gene Structure Display Server (GSDS) website (Hu et al., 2015) (http://gsds.cbi.pku.edu.cn). General Feature Format (GFF) annotations for melons were downloaded from the genome database. The required annotation content for the *CmOMTs* exon-intron structure was uploaded to the GSDS website. The online tool Multiple Em for Motif Elicitation (MEME: https://meme.nbcr.net/) was used to further identify and analyze the conserved protein motifs of the *CmOMT* gene family members, and a motif number of 5 was implemented (Bailey et al., 2009).

### Promoter analysis, transmembrane helix prediction, and structure prediction for CmOMT proteins

The promoter sequences of the *CmOMT* genes were analyzed using PlantCARE (http://bioinformatics.psb.ugent.be/webtools/plantcare/html/). Briefly, we used the sequence 2,000 bp upstream of the ATG start codon of the *CmOMT* gene family as the promoter sequence. *Cis*-regulatory elements in *CmOMT* gene promoters identified using PlantCARE were conserved for visualization using the GSDS website. In standard mode, the three-dimensional (3-D) structure of each CmOMT protein was modeled using the PHYRE2 tool (http://www.sbg.bio.ic.ac.uk/phyre2/html/page.cgi?id=index).

### GO term and expression analysis for *CmOMT* genes

Gene Ontology (GO) term analyses were performed for *CmOMT* genes using the R package (ClusterProfiler package, enrichplot package), and terms with *Q*-values ≤ 0.05 were considered significantly enriched.

The expression of *CmOMT* genes in various organs was examined using a previously published RNA-seq dataset (Yano et al., 2020). A heatmap was generated to illustrate the spatiotemporal expression in the callus, dry seeds, root, stem (downside and upside), shoot apex, leaves (young and 6th–12th), tendril, flower (anther male, petal female, and stigma female), ovary [0–4 DAF (days after flowering)], fruit flesh (8–50 DAF), and fruit epicarp (8–50 DAF). Published RNA-seq datasets analyzed by Sebastiani et al. (2017) and Zhu et al. (2018) were analyzed for *Fusarium oxysporum* f. sp. *melonis* race 1.2 (FOM 1.2) and powdery mildew, respectively. Published RNA-seq data were used to generate heatmaps and volcano plots using the R package (Heatmaply package) and online tool Dynamic volcanogram (https://www.omicshare.com/tools/), respectively. To verify the expression of *CmOMT* genes under different stress conditions, total RNA was extracted using the RNA isolator Total Extraction Reagent (Vazyme, Nanjing, China). The extracted RNA was reverse-transcribed to cDNA using the HiScript II First Strand cDNA Synthesis Kit (Vazyme, Nanjing, China). qRT-PCR assays were performed on a qTOWER^3^G Real-time System (Analytik Jena AG, Jena, Germany). The relative expression based on three biological and technical repeats was calculated by the 2^−ΔΔCT^ method (Livak & Schmittgen, 2002). All primers used for qRT-PCR are listed in Table S1.

### Statistical analysis

All data were analyzed using SPSS software version 26.0 (SPSS Inc., Chicago, IL, USA) and presented as the mean ± standard deviation (SD) of three biological repeats. Differences among the results were tested by one-way ANOVA (one-way analysis of variance), with a *P*-value ≤ 0.05 indicating significance.

## Results

### Identification and characterization of CmOMT genes in *Cucumis melo* L.

**Figure 1:**
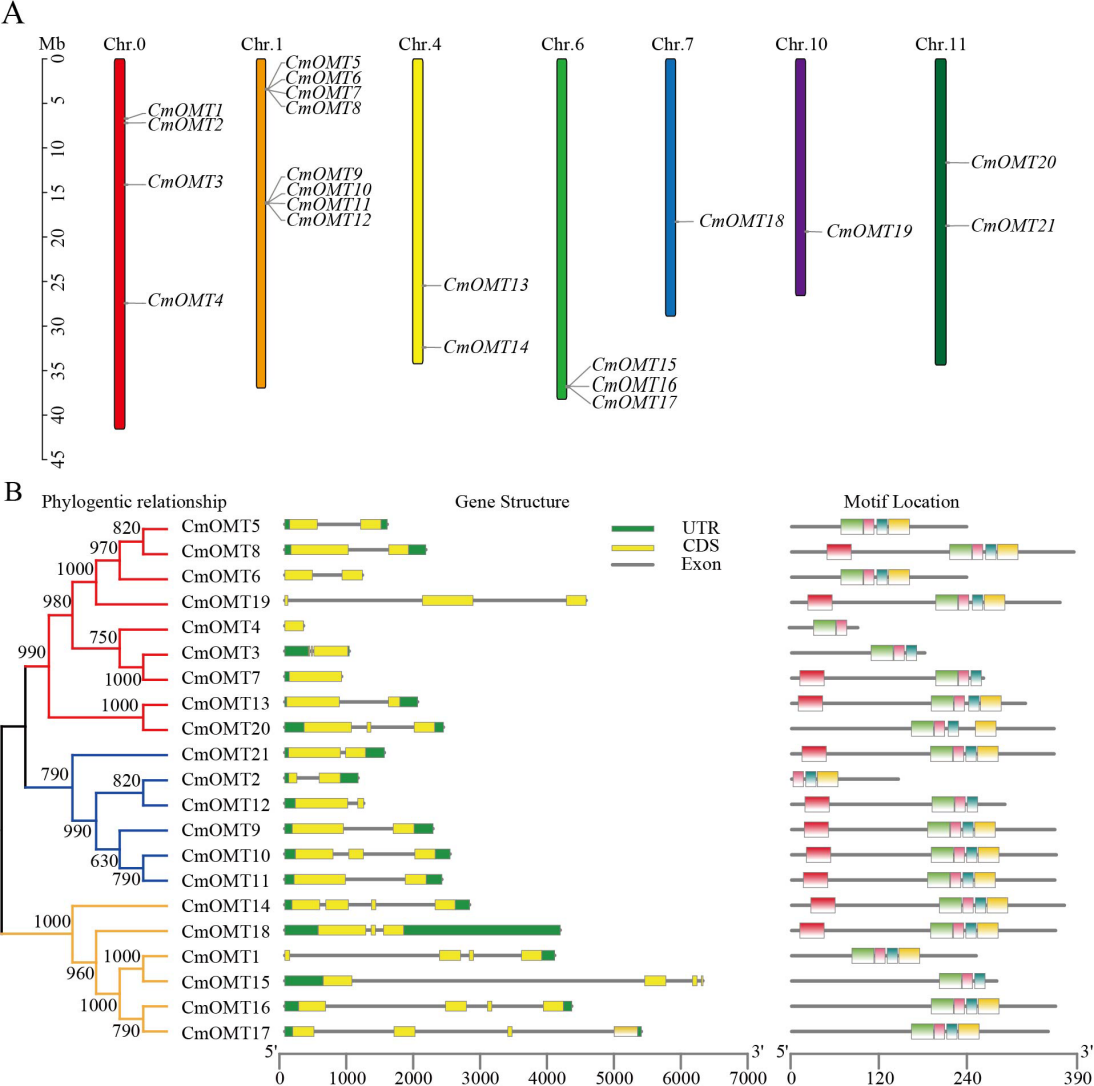
Chromosome distribution of *CmOMTs* (A) and phylogenetic tree, gene structure and conserved motif distribution of CmOMT gene family members (B).

Twenty-one CmOMT candidate genes were identified in the melon genome (Table S2) and named *CmOMT1* to *CmOMT21* based on the chromosomal gene order. Certain *CmOMT* genes were also distributed in adjacent regions on the same chromosome (Table 1 and Fig. 1A). The genes presented an uneven distribution on 12 chromosomes and were present on six chromosomes; however, four *CmOMTs* were not mapped to any of the chromosomes (Fig. 1A). *CmOMTs* were intensively distributed on chromosome 1, three *CmOMTs* were distributed on chromosome 6, two *CmOMTs* were distributed on chromosomes 4 and 11, and only one *CmOMT* gene was distributed on chromosomes 7 and 10 (Fig. 1A). According to a previously defined tandem arrangement, *CmOMT1*–*CmOMT4*, *CmOMT13*, *CmOMT14*, and *CmOMT18*–*CmOMT21* were not tandem duplicated genes, whereas other *CmOMTs* appear to be generated by tandem repeat events (Zhu et al., 2014; Zhao et al., 2018). For example, *CmOMT5*–*CmOMT8* may belong to a tandem duplication gene cluster while *CmOMT9*–*CmOMT12* may be part of another tandem duplication gene cluster.

**Table 1:** The basic information of *CmOMT* genes in melon.

OMT Member	gene ID	Number of amino acids	Molecular weight	PI	Formula	Instability index	Aliphatic index	Gravy
*CmOMT1*	*MELO3C027330*	251	27,891.46	9.13	C_1261_H_1993_N_337_O_352_S_12_	30.79	89.72	−0.139
*CmOMT2*	*MELO3C027370*	145	16,247.76	5.04	C_717_H_1147_N_193_O_216_S_10_	36.73	92.83	−0.094
*CmOMT3*	*MELO3C000487*	181	19,483.45	4.60	C_882_H_1379_N_211_O_266_S_9_	45.16	88.84	0.109
*CmOMT4*	*MELO3C028075*	93	9,555.92	4.47	C_418_H_663_N_107_O_134_S_7_	31.49	79.57	0.280
*CmOMT5*	*MELO3C018855*	238	26,895.11	4.76	C_1225_H_1892_N_296_O_355_S_14_	38.98	86.81	−0.092
*CmOMT6*	*MELO3C018856*	238	26,659.81	5.04	C_1207_H_1883_N_301_O_352_S_13_	37.07	89.71	−0.061
*CmOMT7*	*MELO3C018858*	261	28,713.79	4.59	C_1293_H_1999_N_321_O_391_S_13_	42.33	88.12	−0.002
*CmOMT8*	*MELO3C018859*	384	43,523.50	5.43	C_1966_H_3059_N_501_O_561_S_26_	43.62	90.89	−0.079
*CmOMT9*	*MELO3C013310*	358	40,115.49	5.70	C_1798_H_2831_N_473_O_519_S_23_	42.13	97.82	0.012
*CmOMT10*	*MELO3C013311*	360	39,968.08	5.47	C_1775_H_2802_N_466_O_531_S_25_	42.99	90.97	−0.061
*CmOMT11*	*MELO3C013313*	358	39,806.86	5.60	C_1769_H_2807_N_481_O_522_S_20_	41.38	98.32	−0.002
*CmOMT12*	*MELO3C013315*	290	32,129.94	5.25	C_1447_H_2275_N_373_O_428_S_12_	31.14	97.83	−0.002
*CmOMT13*	*MELO3C026750*	318	35,978.56	5.52	C_1608_H_2527_N_423_O_470_S_21_	58.95	92.58	−0.172
*CmOMT14*	*MELO3C009403*	371	40,926.88	5.84	C_1814_H_2860_N_490_O_545_S_21_	41.61	84.93	−0.161
*CmOMT15*	*MELO3C014089*	279	30,768.32	5.36	C_1379_H_2167_N_351_O_416_S_14_	36.72	92.62	0.002
*CmOMT16*	*MELO3C014091*	359	39,670.30	5.14	C_1780_H_2775_N_463_O_533_S_15_	33.62	92.84	−0.030
*CmOMT17*	*MELO3C014098*	349	38,759.51	5.41	C_1742_H_2709_N_457_O_508_S_18_	20.07	92.15	−0.004
*CmOMT18*	*MELO3C024861*	359	39,323.66	5.89	C_1774_H_2778_N_458_O_506_S_22_	30.82	96.43	0.091
*CmOMT19*	*MELO3C018336*	365	41,114.67	5.84	C_1859_H_2909_N_483_O_528_S_20_	43.08	95.07	−0.089
*CmOMT20*	*MELO3C019324*	357	40,254.50	5.90	C_1801_H_2852_N_472_O_531_S_20_	45.21	93.42	−0.148
*CmOMT21*	*MELO3C013600*	357	39,648.44	5.45	C_1793_H_2769_N_461_O_522_S_16_	42.55	92.66	−0.045

The chromosome location, strand, description, number of amino acids, molecular weight, PI, formula, instability index, aliphatic index, and grand average hydropathicity (GRAVY) characteristics of the CmOMT genes were obtained. The average amino acid sequence of the 21 CmOMT proteins was 298 amino acids, with the value ranging from 93 (CmOMT4) to 384 amino acids (CmOMT8) (Table 1). The average molecular weight of the 21 CmOMT proteins was 33,211.24, the PI was approximately 5.50, the instability index was approximately 38.88, and the aliphatic index was approximately 91.63 (Table 1). In addition, five of the CmOMT proteins (CmOMT3, CmOMT4, CmOMT9, CmOMT15, and CmOMT18) were hydrophobic (Table 1).

### Analysis of the gene structure and motifs in *CmOMT* genes

To further investigate the *CmOMT* gene family, the UTR and CDS distribution and number of *CmOMT* genes were analyzed based on phylogenetic trees. On the same branch, the CmOMTs were divided into class I and class II, which contained nine and six CmOMTs, respectively (Fig. 1B). On the other branch, six CmOMTs were grouped into class III, which was distinct from classes I and II (Fig. 1B). Genetic structural characterization showed that the number of CDS in classes I and II was similar, with 1–3 CDS regions (Fig. 1B). Class III differed from classes I and II in terms of gene structure and included 4 CDS regions, excluding *CmOMT18* (Fig. 1B). Each exon in the same sister gene pair was very similar and presented the same size. However, introns were more variable in sequence length than the strongly conserved exons of sister gene pairs. This result indicates that introns are evolutionarily more unstable than exons, which is consistent with traditional evolutionary theory (Zhang et al., 2021a; Zhang et al., 2021b).

Conserved protein motifs were identified in CmOMTs. Five different motifs with 15–34 amino acids were found in the CmOMT gene family (details on the conserved structural domains are illustrated in Fig. S1A). CmOMT protein sequences were compared using a phylogenetic tree, and the locations were mapped using clustering and motif analyses. Details of the CmOMTs are presented in Fig. S1B. Motif 1 was present in most of the CmOMTs except for CmOMT2 (Fig. 1B). Motif 2 was not present in CmOMT3, CmOMT4, CmOMT7, CmOMT12, and CmOMT15 (Fig. 1B). Motifs 3 and 4 represented the basis of the CmOMT structural domain because all CmOMTs contained motifs 3 and 4. Motif 5 was identified in only nine CmOMT members, which belonged to class I (CmOMT7, CmOMT8, CmOMT13, and CmOMT19) and class II (CmOMT9–CmOMT12 and CmOMT21) (Fig. 1B). These results suggested that there were significant differences between CmOMT proteins after whole genome duplication (WGD) events and that the loss and retention of motifs may be related to the evolution of subbranches.

### Phylogenetic tree and structure analysis of OMT proteins

**Figure 2:**
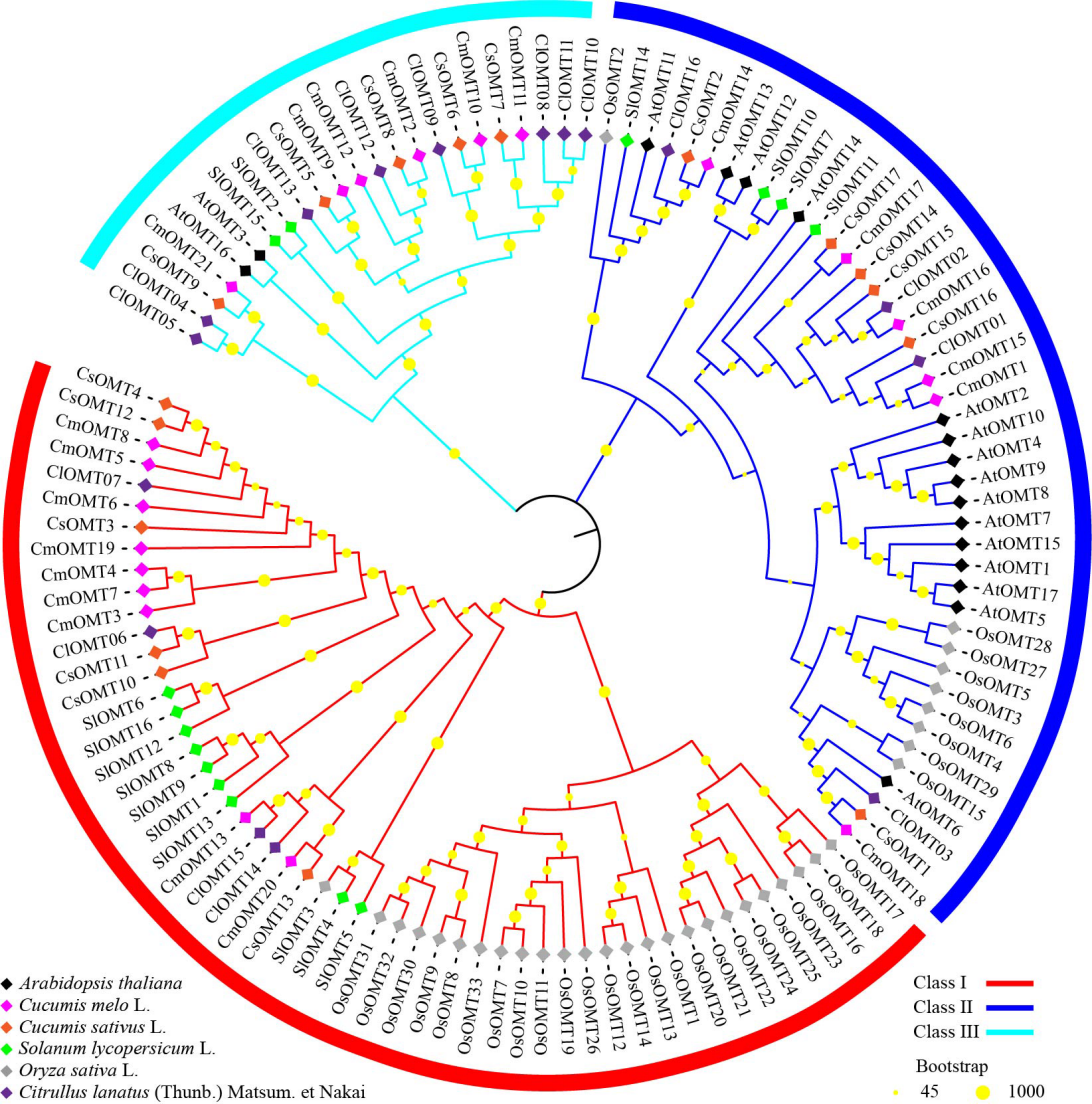
Phylogenetic analysis of *OMTs* from *Arabidopsis thaliana*, *Cucumis melo* L., *Cucumis sativus* L., *Solanum lycopersicum* L., *Oryza sativa* L., and *Citrullus lanatus* (Thumb.).

To investigate the evolutionary relationships of plant OMT homologs, 120 OMTs were identified in melon, *Arabidopsis* (Chen et al., 2021), cucumbers (Table S3), tomatoes (Lu et al., 2019), rice (Liang et al., 2022) and watermelon (Chang et al., 2021), and an unrooted phylogenetic tree was constructed using the NJ algorithm. The phylogenetic tree branches showed that OMTs from the selected plant species could be divided into class I, class II, and class III, which contained 53, 44, and 23 OMTs, respectively (Fig. 2). Class I contained nine CmOMTs, six CsOMTs, ten SlOMTs, 24 OsOMTs, and four ClOMTs, and it was not included among the AtOMTs (Fig. 2). Class II contained six CmOMTs had 15 AtOMTs, six CsOMTs, four SlOMT genes, nine OsOMTs, and four ClOMTs (Fig. 2). Class III did not contain OsOMTs and was composed of six CmOMTs, two AtOMTs, five CsOMTs, two SlOMTs, and eight ClOMTs (Fig. 2). Multiple species form gene clusters at the base of the NJ tree, with melon, cucumber, tomato, and watermelon showing a closer homology of OMT proteins than *Arabidopsis* and rice, which may reflect the functional diversification of the OMT gene family after the evolution of dicotyledonous and monocotyledonous plants. In addition, cucurbit OMT proteins (CmOMTs, CsOMTs, and ClOMTs) showed a close homology, suggesting that the OMT family experienced species-specific amplification during the evolutionary process.

The structure of a protein determines its function. Therefore, the 3-dimensional structures of CmOMTs were predicted using the PHYRE2 tool using the standard mode. Different percentages of alpha-helices, beta-sheets, and TM-helices were found in the CmOMTs (Fig. S2). Nine CmOMTs (CmOMT7, CmOMT8, CmOMT10, CmOMT12–CmOMT15, CmOMT19, and CmOMT21) had the highest proportions of alpha helices (Fig. S2, Table S4). CmOMT2, CmOMT5 and CmOMT6 contained the highest number of beta-sheets, only CmOMT8, CmOMT9, CmOMT11, CmOMT14, CmOMT16, CmOMT18, and CmOMT21 contained TM-helixes (Fig. S2, Table S4). In addition, disordered regions (DRs) were found in all CmOMTs, and these regions warrant further exploration (Fig. S2, Table S4).

### Synteny and GO enrichment analyses of *CmOMT* genes

Synteny analysis of *CmOMTs* was performed using the MCScan tool, and the fragment tandem duplication events of the *CmOMT* gene family members were investigated. As shown in Fig. S3A, only one *CmOMT* was involved in fragment tandem duplication events among the *CmOMT* gene family (Table S5). These results suggest that few *CmOMTs* arose from fragment tandem duplication events; thus, fragment tandem duplication events have a limited evolutionary role in *CmOMTs*. To further explore the phylogenetic mechanisms of *CmOMTs*, we constructed syntenic maps of melon based on three species, including a dicot (*Arabidopsis thaliana*) and two monocots (*Citrullus lanatus* and *Cucumis sativus*) (Fig. S3). Multispecies syntenic analysis showed that 2, 6, and 5 *CmOMT* genes had syntenic relationships with *Arabidopsis*, watermelon, and cucumber, respectively (Fig. S3). Collinear *CmOMT9* and *CmOMT14* gene pairs were found between melon and *Arabidopsis* (Fig. S3B, Table S5). Six collinear *CmOMT* gene pairs (*CmOMT5*/*9*/*13*/*14*/*20*/*21*) were identified between melons and watermelons (Fig. S3C, Table S5), five collinear *CmOMT* gene pairs (*CmOMT5*/*9*/*14*/*18*/*21*) were associated with cucumber (Fig. S3C, Table S5). Collinear *CmOMT9* and *CmOMT14* gene pairs were identified between melon and *Arabidopsis*/watermelon/cucumber, indicating that homologous gene pairs may have been generated before the divergence of monocotyledonous and dicotyledonous plants. Collinear *CmOMT5* and *CmOMT18* gene pairs were found between melon and other Cucurbitaceae species, suggesting that these *OMT* genes may have played key roles in the evolution of Cucurbitaceae *OMTs*.

To obtain a comprehensive understanding of the gene functions, GO term analyses were performed for all *CmOMTs*. The results of the GO enrichment analysis (*Q*-value ≤ 0.05) showed the genes corresponded to two biological process classes, namely, ‘methylation’ (*Q*-value = 6.92e−32) and ‘metabolic process’ (*Q*-value = 0.0055), and 11 molecular function classes (Table S6), which were closely associated with methyltransferase, such as ‘O-methyltransferase activity’ (*Q*-value = 2.6e−58), ‘methyltransferase activity’ (*Q*-value = 1.25e−33), and ‘caffeate O-methyltransferase activity’ (*Q*-value = 0.0016) (Table S6). These findings were consistent with previous reports showing that the *OMT* gene family is associated with methylation processes in plants (Liu et al., 2015).

### *Cis*-acting element analysis of *CmOMT* genes in promoter regions

**Figure 3:**
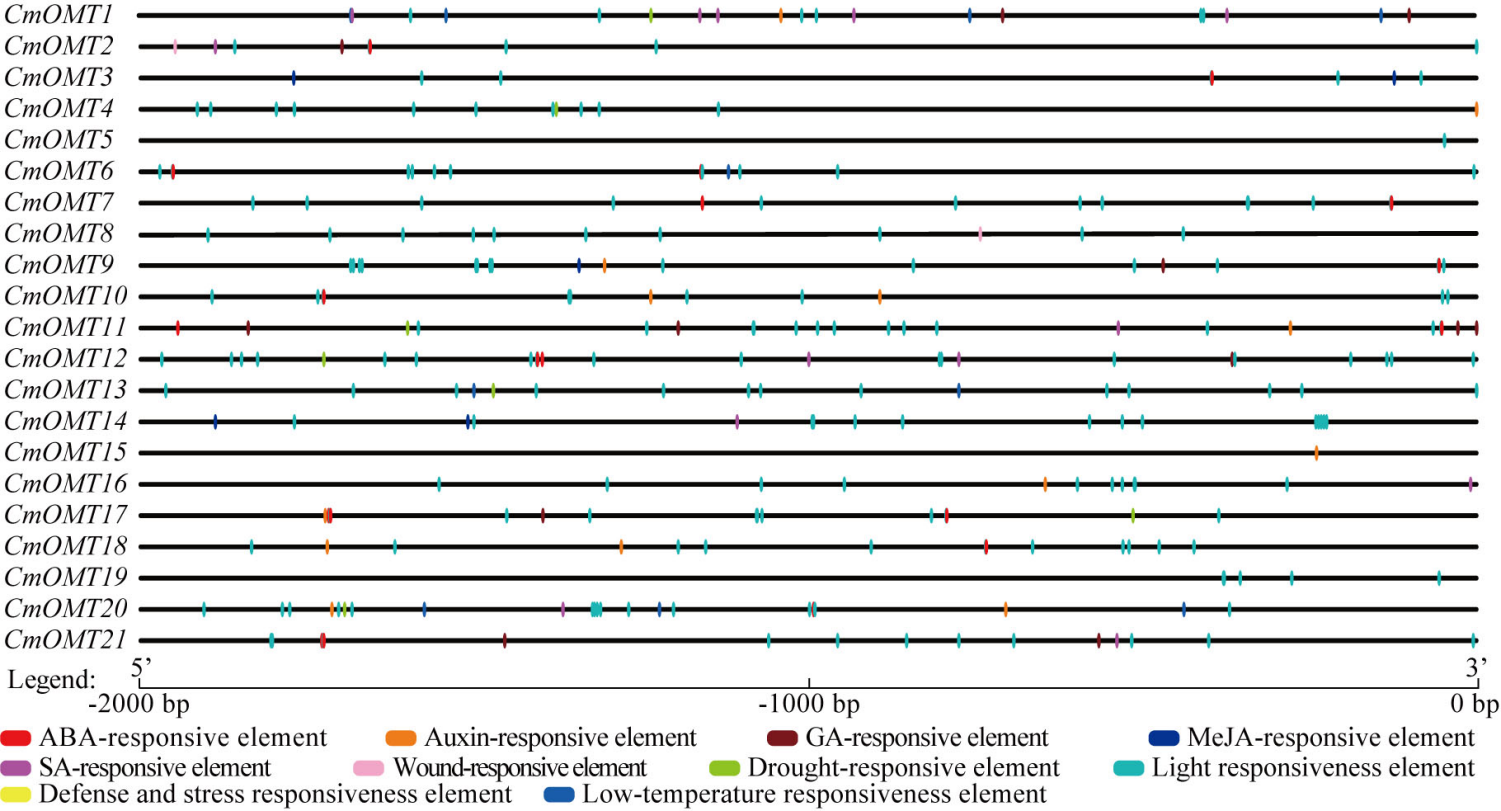
Description of *cis*-elements in *CmOMT* promoters. Different colors represent different *cis*-acting elements. Ten *cis*-acting elements (ABA, auxin, GA, MeJA, SA, wound, drought, light, defense, stress and low-temperature respon.

*Cis*-acting elements are transcription factor-specific binding sites that play important roles in regulating plant growth, differentiation, and development. We extracted a 2,000 bp sequence in the upstream region of each *CmOMT* and used the PlantCARE tool to identify the *cis*-acting elements in *CmOMT* promoters. Based on functional labeling, 10 *cis*-acting elements were obtained, among which 50% (5/10) was associated with hormonal responses and 50% was associated with stress and developmental responses (Fig. 3 and Table S7). Thirteen, ten, and seven *CmOMT* promoters contained ABA-responsive elements (ABREs), auxin-responsive elements (AuxRR-core and TGA-element), and GA-responsive elements (P-box, TATC-box, and GARE-motif), respectively (Fig. 3 and Table S7). MeJA-responsive elements (CGTCA-motif and TGACG-motif) and SA-responsive elements (TCA-element) were present in nine and eight *CmOMTs* promoters, respectively (Fig. 3 and Table S7). Wound-responsive elements (WUN-motifs) were present only in *CmOMT2* and *CmOMT8* promoters (Fig. 3 and Table S7). A drought-responsive element (MBS), defense and stress responsiveness element (TC-rich repeats), and low-temperature responsiveness (LTR) element were observed in 7, 8, and 4 *CmOMT* promoters (Fig. 3 and Table S7). The light-responsiveness element was commonly found in the promoters of all *CmOMTs*.

### Expression analysis of *CmOMT* genes in different tissues and developmental stages

**Figure 4:**
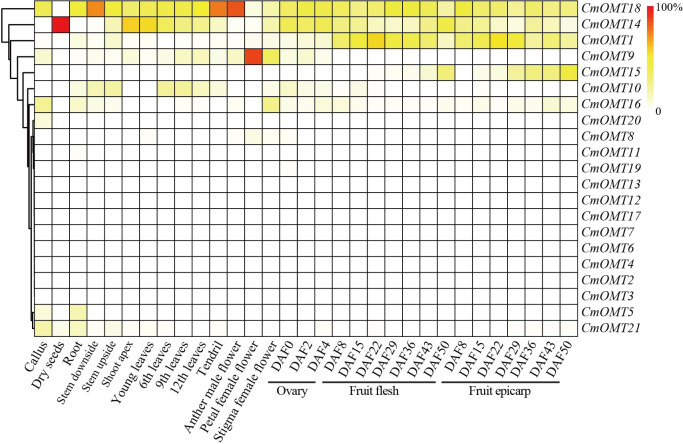
Gene expression heatmap was represented by scaling expression of *CmOMT* gene across samples under normal growth conditions. Published RNA-seq datasets were used to evaluate *CmOMTs* (Yano et al., 2020).

Our results revealed that a large number of development- and stress-related *cis*-acting elements were widely distributed in *CmOMTs*. We further explored the expression profiles of *CmOMTs* in different tissues and developmental stages. Gene expression profiling data were used to analyze the expression of *CmOMTs* in different tissues and at different developmental stages, and a gene expression heatmap was presented by scaling the expression of each gene across samples (Fig. 4), including the callus, dry seeds, root, stem (stem downside and upside), shoot apex, leaves (young, 6th, 9th and 12th leaves), tendril, flower (anther in male, petal in female, and stigma in female flowers), ovary (DAF 0, 2 and 4), fruit flesh (DAF 8, 15, 22, 29, 36, 43 and 50), and fruit epicarp (DAF 8, 15, 22, 29, 36, 43 and 50) (Table S8). Three *CmOMTs* (*CmOMT16*/18/21) were highly expressed in the callus, and only *CmOMT14* was expressed in dry seeds. Nine *CmOMTs* were highly expressed in the roots, *e.g.*, *CmOMT5*/*18*/*21*. Seven *CmOMTs* were highly expressed in the stems, with *CmOMT1*/10/18 showing high expression in both the downside and upside of stems. *CmOMT9*/10/14/18 was highly expressed in leaves (young, 6th, 9th, and 12th leaves), tendrils, and flowers (anther in male, petal in female and stigma in female flower). Notably, *CmOMT9* was expressed at higher levels in the petals of female flowers than the other *CmOMTs* (Fig. 4 and Table S8). *CmOMTs* that play a key role in fruit pigment methylation can affect plant coloration and improve plant self-protection against environmental stress (Roldan et al., 2014; Du et al., 2015). In the ovary (DAF 0, 2, and 4), *CmOMT14*/*18* was highly expressed. *CmOMT1*/*18* showed an increasing and then decreasing trend during fruit flesh ripening (DAF 8, 15, 22, 29, 36, 43, and 50), consistent with the fruit epicarp. *CmOMT14*/*15* showed an overall increasing trend in fruit flesh (DAF 8, 15, 22, 29, 36, 43, and 50), *CmOMT14* displayed an increasing and then decreasing trend during fruit flesh ripening (Fig. 4 and Table S8). Overall, *CmOMT1*/*14*/*15*/*18* were highly expressed throughout the growth and developmental stages of melon and in most tissues.

### Expression of *CmOMT* genes in response to abiotic and biotic stresses

**Figure 5:**
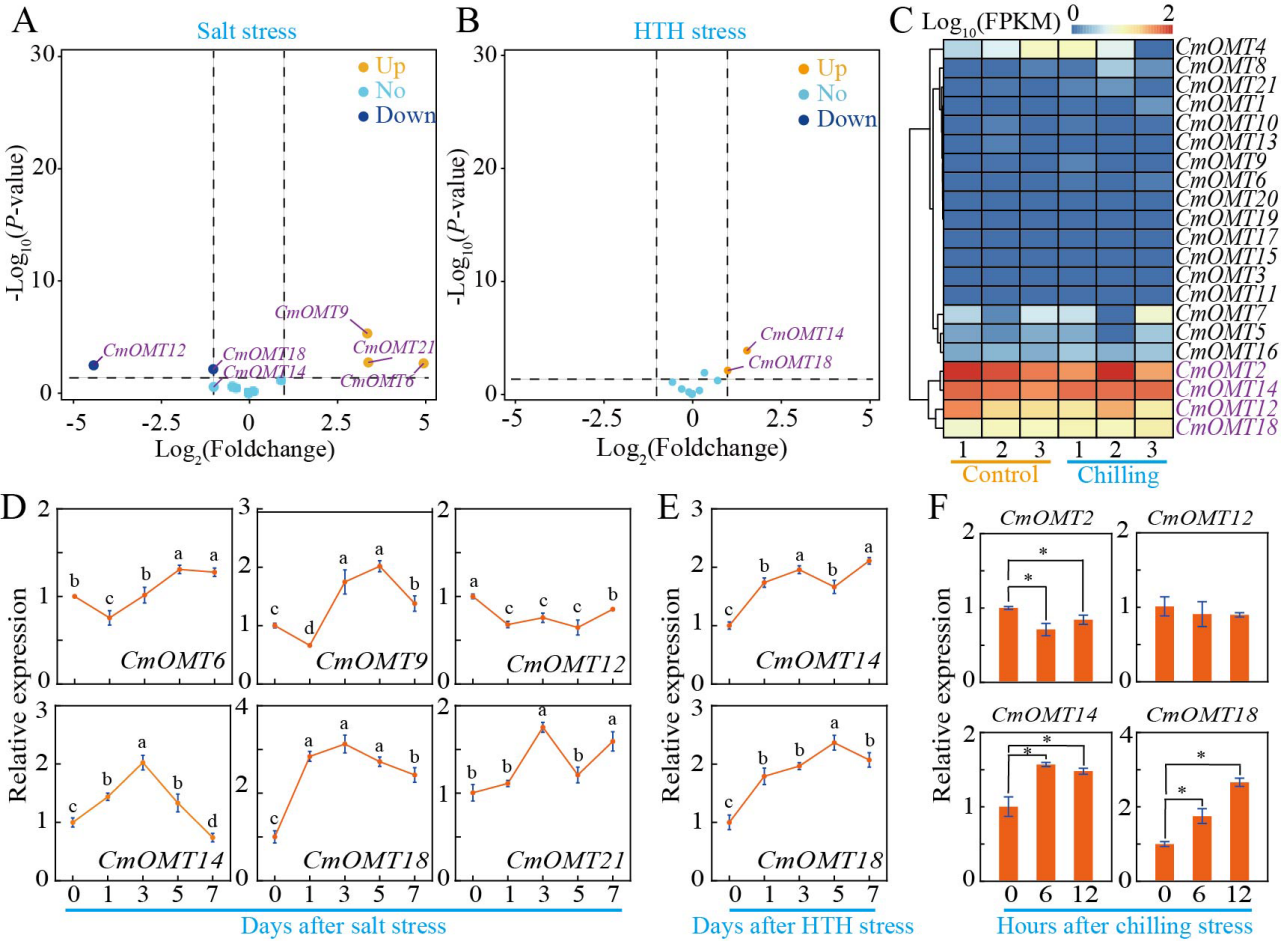
Expression profiles of *CmOMT* gene family under multiple abiotic stresses. Volcano plot of 21 *CmOMTs* in response to salt (A) and HTH (B) stresses based on public RNA-seq data (Wang et al., 2016; Weng et al., 2022). Heatmap of 21 *CmOMTs* in response to chilling stress (C) based on public RNA-seq data (Diao et al., 2020). qRT-PCR validation of differential expression of *CmOMTs* involved in salt (D), HTH (E), and chilling (F) stresses. Asterisks and different letters indicate statistically significant differences (*P* < 0.05).

To investigate whether *CmOMTs* are involved in abiotic and biotic stress responses, we analyzed the expression patterns of 21 *CmOMTs* in varieties with different stress tolerances based on publicly available transcriptomic and qRT-PCR data. The stressors included salt (300 mM NaCl), HTH, cold (6 °C), *Fusarium oxysporum* f. sp. *melonis* race 1.2 (FOM1.2), and powdery mildew (Wang et al., 2016; Sebastiani et al., 2017; Zhu et al., 2018; Diao et al., 2020; Weng et al., 2022). The results showed that the expression levels of *CmOMT6/9/12/14/18/21* were more variable after salt stress, which could be expanded on in a future study (Fig. 5A). The qRT-PCR results showed that *CmOMT6* and *CmOMT9* were initially downregulated and then upregulated within 7 d of salt stress, *CmOMT12* was significantly downregulated, and *CmOMT14/18/21* showed a peak in expression at 3 d after salt stress (Fig. 5D). In summary, *CmOMT6/9/14/18* may have a positive regulatory role in response to salt stress, *CmOMT14/18* may play a pivotal role in the early stages of salt stress, and *CmOMT12* may have a negative regulatory role in response to salt stress. Based on transcriptome data, the results revealed that *CmOMT14/18* was highly expressed after HTH stress (Fig. 5B). RT-qPCR analysis indicated that the expression of *CmOMT14/18* was induced by HTH stress and that *CmOMT14/18* were consistently upregulated after HTH stress (Fig. 5E). Of the 21 *CmOMTs* that responded to cold stress, four *CmOMTs* (*CmOMT2/12/14/18*) were highly expressed under both the control and cold stress conditions, suggesting that these genes may play a potential role in the mitigation of cold stress (Fig. 5D). RT-qPCR analysis revealed that the expression pattern of *CmOMT2* was significantly decreased after cold stress and that the expression pattern of *CmOMT14/18* was significantly upregulated after cold stress, indicating that these genes may have a positive regulatory role after cold stress (Fig. 5F). Overall, the transcript levels of *CmOMT14/18* were significantly affected by multiple abiotic stressors, suggesting a positive regulatory role.

**Figure 6:**
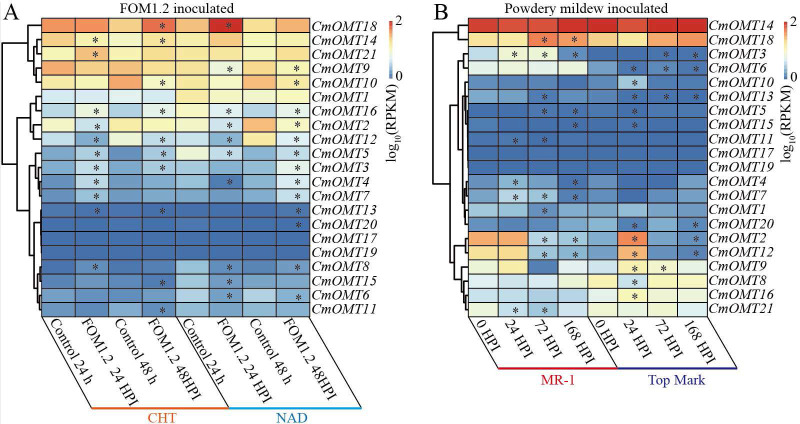
Expression pattern of the *CmOMT* gene family in response to FOM1.2 (A) and powdery mildew (B). Asterisks (*) indicate statistically significant differences (*P* < 0.05).

To explore the potential function of the 21 *CmOMTs* in response to biotic stress, published RNA-seq data were used to analyze the expression pattern of the *CmOMT* gene family under FOM1.2 and powdery mildew infection (Sebastiani et al., 2017; Zhu et al., 2018). Fifteen *CmOMTs* (*CmOMT2/3/4/5/7/8/10/11/12/13/14/15/16/18/21*) were significantly changed in ‘CHT’ (susceptible cultivar) after FOM1.2 infection, among which seven *CmOMTs* (*CmOMT3/4/5/7/8/16/21*) were significantly upregulated at 24 hpi (hours post FOM1.2 inoculation) and five (*CmOMT3/5/14/16/18*) were significantly upregulated at 48 hpi (Fig. 6A). Similarly, 15 *CmOMTs* (*CmOMT2/3/4/5/6/7/8/9/10/12/13/15/16/18/20*) were significantly up/downregulated in the resistant cultivar ‘NAD’ after FOM1.2 inoculation, only one (*CmOMT18*) was upregulated at 24 hpi, and eight (*CmOMT3/4/5/7/8/13/16/20*) were upregulated at 48 hpi (Fig. 6A). Interestingly, *CmOMT18* reacted to FOM1.2 at an earlier stage in ‘NAD’ than ‘CHT,’ which showed changes at 48 hpi (Fig. 6A). Therefore, *CmOMT18* may have a greater potential for FOM1.2 resistance. In addition, to verify whether the *CmOMT* gene family was involved in the response to FOM1.2, a correlation analysis was performed between resistance genes (*PR-1* and *LRR* genes) and *CmOMT* s. *PR-1* was positively correlated with *CmOMT18*, and most *LRR* genes showed a favorable positive correlation with *CmOMT18* (Fig. S4). Taken together, *CmOMT18* may play a positive regulatory role in the response to FOM1.2. To examine the reaction of the *CmOMT* gene family to powdery mildew, six *CmOMTs* (*CmOMT3/4/5/7/11/18*) were upregulated in MR-1 (resistant cultivar) after powdery mildew inoculation, with *CmOMT3/7* upregulated at 1 and 3 dpi (days post-powdery mildew inoculation) and *CmOMT5/18* upregulated at 3 and 7 dpi. Most *CmOMTs* were up/downregulated in the susceptible cultivar ’Top mark’ after powdery mildew inoculation: *CmOMT2/9/10/12/15/16* were upregulated at 1 dpi, *CmOMT3/9* was upregulated at 3 dpi, and *CmOMT3* was upregulated at 7 dpi (Fig. 6B). As *CmOMT18* was specifically expressed in leaves, a correlation analysis of the *CmOMT* gene family with *R* genes revealed that *CmOMT18* may be involved in the regulation of melon resistance to powdery mildew (Fig. S4).

## Discussion

Plants are pedicellates and therefore cannot escape from unfavorable environmental conditions that can affect their growth and development during their life cycle. Unfavorable stress conditions, such as abiotic and biotic stresses, are considered major environmental stressors capable of limiting plant growth and development and directly affecting agricultural yields (Batista-Silva et al., 2019; Yuan et al., 2021). Previous studies have found that secondary metabolites are involved in a variety of biological processes (plant growth, development, and environmental response) and that OMT can catalyze the O-methylation of a variety of secondary metabolites (Liu et al., 2015). *OMT* genes are widely present in plants such as *Arabidopsis*, rice, tomato, and watermelon (Lu et al., 2019; Chang et al., 2021; Liang et al., 2022). In this study, we performed a genome-wide prediction of *CmOMTs* based on the simultaneous detection of conserved OMT structural domains using HMMER, CDD, and SMART tools. We identified 21 *CmOMTs*, which were named *CmOMT1*–*CmOMT21*, in *Arabidopsis* (17), watermelon (16), and *Citrus* (58) (Lu et al., 2019; Chen et al., 2021; Chang et al., 2021). Genomic DNA, CDS length, and deduced amino acid sequences varied among the CmOMT genes, leading to variations in the theoretical molecular weight, PI, instability index, aliphatic index, and GRAVY of CmOMTs (Table 1). *CmOMTs* were unevenly distributed on all chromosomes, with most *CmOMTs* on chromosome 1 (Fig. 1 and Table 1). Similar *CmOMT* distribution patterns have been observed in other plants, such as *Arabidopsis*, watermelon, *Citrus*, and rice (Lu et al., 2019; Chen et al., 2021; Chang et al., 2021; Liang et al., 2022).

The phylogenetic relationship analysis showed that OMT genes could be clustered within a monophyletic group derived from non-plant genes. Based on functional traits, the results indicated the existence of two plant serial group clusters (Lam et al., 2007). In this study, 120 OMT proteins were divided into three branches. Although different from the evolutionary analysis results of other species, the OMT genes of cucurbit crops, such as watermelon, were similarly divided into three categories, which may be due to tandem duplication (Lu et al., 2019; Chang et al., 2021; Chen et al., 2021; Liang et al., 2022). The presence of ancient duplication events and high retention rates has led to the existence of a large number of duplicated genes in plant genomes. These duplicated genes have contributed to the evolution of genes with novel functions, such as the production of floral structures, enhanced disease resistance, and adaptation to different adversities (Nicholas, Melissa & Shin-Han, 2016; Zhang et al., 2020). Cucurbitaceae OMT proteins may have undergone more favorable changes during evolution, thereby generating new functions (Chang et al., 2021). Of the three branches, class I had the most OMT members, with 53 OMT proteins, although *Arabidopsis* had nine CmOMTs, four ClOMTs, ten SlOMTs, six CsOMTs, and 24 OsOMTs. These findings were similar to the results of a study on watermelon (Chang et al., 2021). Differences in the phylogenetic relationships between plant OMT homologous proteins may reflect the diversity of their functions in growth, development, and environmental responses. In *Arabidopsis*, the OMT protein AtOMT3 is a class III protein that has close homology with CmOMT9. AtOMT3 is defined as AtASMT1, which increases melatonin levels, improves plant salt tolerance, and is closely related to *Pst DC3000* resistance (Shi, Wei & He, 2016; Zheng et al., 2017). AtOMT6 is an OMT protein on class II OMT protein that shows close homology with CmOMT8 and has been identified as AtCOMT1, which is associated with melatonin synthesis, regulating melatonin synthesis levels, and improving resistance to multiple stresses in plants, such as salt stress and heat stress (Zheng et al., 2017; Hasan et al., 2023). In contrast, Chang et al. (2021) reported that *ClCOMT1* (*ClOMT3*) is also closely homologous to *CmOMT18* and could enhance abiotic stress resistance in *Arabidopsis* by increasing endogenous melatonin content. For class I OMT proteins, Liang et al. (2022) reported that OsCOMT8, OsCOMT9, and OsCOMT15 play key roles in lignin synthesis. Furthermore, we observed that CmOMTs presented a closer relationship with the OMTs of watermelon, cucumber, and tomato than those of *Arabidopsis* and rice in each branch. This finding implies that the *OMT* gene family of dicotyledons and monocotyledons experienced functional diversification during their long-term evolution. This hypothesis is partially supported by the observation that OMT transcript abundance and expression patterns in *Arabidopsis* and rice differ significantly from those in watermelon and melon under normal growth conditions (Lu et al., 2019; Chen et al., 2021; Chang et al., 2021; Liang et al., 2022).

One of the relatively reliable parameters for evaluating the evolution of gene families is variation in structure or motifs. Analyses of plant evolutionary relationships have shown that genes with similar intron-exon structures and conserved motif arrangements often have similar functions (Long, Souza & Gilbert, 1995). Most members of the same taxon of the 67 *OMTs* in soybean have similar gene structures, and the same results have been observed in pomegranate and rice (Zhang et al., 2021a; Zhang et al., 2021b; Zhao et al., 2022; Liang et al., 2022). In our study, 21 *CmOMTs* were classified as class I, class II, and class III, which consisted of nine, six, and six members, respectively. This classification is consistent with observations in watermelons (Chang et al., 2021) and is further supported by the fact that cucurbits are different from other species. Analysis of the differences in exon-intron patterns of *CmOMTs* among the three classes showed that most of class I contained 1–2 exons, class II contained 2–3 exons, class III contained more than three exons, and the intron region was longer in class III. Similar exon-intron patterns within the same phylogenetic class may be associated with the tandem duplication of these sequences (Du et al., 2015).

GO enrichment and *cis*-acting element analyses are important methods for gene function studies and have been widely used to predict the possible biological functions and regulatory patterns of gene sets of interest in organisms (Wang et al., 2022). We performed GO enrichment analysis of the *CmOMTs* and identified a total of 13 overrepresented terms (*Q*-value ≤ 0.05), and they were mainly related to methylation in the biological process category and methyltransferase activity in the molecular function category. The results were similar to the functions of *OMTs* in soybean and watermelon, thus demonstrating the similar functions of *OMTs* in different species (Chang et al., 2021; Zhao et al., 2022). The specific binding of *cis*-acting elements with transcription factors in the promoter to regulate gene expression is not only the most important method of biological signal transduction but also an important means by which genes interact with other genes (Cheng et al., 2019). To better understand the role of *CmOMTs* in the response to abiotic and biotic stresses, we analyzed the type, number, and distribution of *cis*-acting elements of 21 *CmOMTs* in the promoter. Light-responsive elements were present in all *CmOMTs*, suggesting that the expression of *CmOMTs* may be regulated by light, similar to previous studies on soybeans (Zhao et al., 2022). In addition, the number of hormone-response *cis*-acting elements and stress-response *cis*-acting elements suggests that *CmOMT* family members may be extensively involved in growth, development, and environmental stress. *OMTs* have been associated with enhanced salt stress resistance in *Arabidopsis*, tomatoes, and watermelons (Chun et al., 2019; Liu et al., 2019; Chang et al., 2021). Moreover, they are reactive to drought and low-temperature stress (Sun et al., 2019; Chang et al., 2021) and involved in disease resistance, including *Pst* DC3000 and *Xoc* (Zhao et al., 2015; Ahammed et al., 2020).

Gene duplication has occurred in 70–80% of angiosperms and represents a method of generating new genes and responding to environmental stress (Panchy, Lehti-Shiu & Shiu, 2016; Qiao et al., 2019). Five pairs of duplicated genes out of the 21 *CmOMTs* suggested that tandem duplication events greatly contributed to the expansion of the *CmOMT* gene family and a similar result was also obtained for tomato (Sun et al., 2021). To examine the phylogenetic relationships of *OMT* genes between melon and other plants, collinear relationships between melon and *Arabidopsis*, watermelon, and cucumber were investigated. The number of collinear events between melon and watermelon and cucumber was much greater than that between melon and *Arabidopsis*, which is consistent with the smaller evolutionary distance between genera of the same family (Sun et al., 2021).

Analyses of gene expression profiles in plants can reveal the functions of genes. For example, *ClOMT7* is expressed only in the root while *OsCOMT7* is expressed at the highest level in the stem (Liang et al., 2022; Chang et al., 2021). Moreover, the expression patterns of *OMT* genes vary among different species. Therefore, our tissue- and development-specific expression profiles of *CmOMTs* provide a basis for determining the involvement of *CmOMTs* in plant physiological and biochemical activities. Previous studies showed that *OMT* genes play important roles in abiotic and biotic stress responses and are key enzymes for the synthesis of lignin and the regulation of plant physiological responses to stresses, such as drought, salt, and high or low temperatures (Hamada et al., 2004) They also play a role in plant resistance to pathogenic invasion by regulating the synthesis of secondary metabolites (Maury, Geoffroy & Legrand, 1999; Zhao et al., 2004; Kim et al., 2007). Analysis of transcriptome and RT-qPCR data under salt, low temperature, and HTH stresses showed that seven *CmOMTs* were involved in the stress response to different degrees. The *CmOMT* gene family plays an important role in some abiotic stress responses, among which the transcript levels of *CmOMT14* and *CmOMT18* showed an increasing trend under all three stress conditions, indicating that they play important roles under abiotic stress conditions. Fifteen *CmOMTs* were significantly altered under FOM1.2 infection, and 12 *CmOMTs* were significantly upregulated under powdery mildew infection in both resistant and susceptible melon varieties. These results suggest a role for *CmOMTs* in the response to certain biotic stresses. The correlation of *CmOMT* genes with *R* genes showed that *CmOMT18* was more correlated with *R* genes under FOM1.2 and powdery mildew infection. The OMT phylogeny analysis among plant species also indicated that *CmOMT18* is closely homologous to *AtOMT16* (*AtCOMT1*) and *ClOMT3* (*ClCOMT1*) and *CmOMT18* may be related to *AtOMT6* (*AtCOMT1*) and *ClOMT3* (*ClCOMT1*) based on the homology among proteins under biotic and abiotic stress (Zheng et al., 2017; Chang et al., 2021).

## Conclusions

In conclusion, *OMT* gene families of melon and five other representative plants were investigated in this study to identify their roles in biotic and abiotic stress. We identified the chromosomal locations, phylogenetic relationships, gene structures, and conserved motifs of *CmOMTs* and determined the expression patterns of *CmOMTs* in response to abiotic and biotic stresses *via* RNA-seq and RT-qPCR. These results not only provide new insights into the characteristics of the plant *OMT* family but will also facilitate functional genomic investigations of *CmOMT* genes in future studies.

## Supplemental Information

10.7717/peerj.16483/supp-1 Supplemental Information 1 List of quantitative real-time PCR (qRT-PCR) primers for expression analysis of CmOMT genes

10.7717/peerj.16483/supp-2 Supplemental Information 2 Identification of conserved domains in CmOMT proteins using the HEMMER tool

10.7717/peerj.16483/supp-3 Supplemental Information 3 Information about OMT proteins in Cucumis sativus L

10.7717/peerj.16483/supp-4 Supplemental Information 4 3-D structures of CmOMT proteins predicted using the PHYRE2 tool

10.7717/peerj.16483/supp-5 Supplemental Information 5 Orthologous relationships between melon and Arabidopsis/watermelon/cucmber

10.7717/peerj.16483/supp-6 Supplemental Information 6 List of GO (gene ontology) terms of CmOMT genes

10.7717/peerj.16483/supp-7 Supplemental Information 7 Identification of cis-regulatory elements in the promoters of CmOMT genes

10.7717/peerj.16483/supp-8 Supplemental Information 8 Tissue space expression profile of CmOMT genes under normal growth conditions Published RNA-seq datasets were used to evaluate CmOMT genes (Yano et al., 2020).

10.7717/peerj.16483/supp-9 Supplemental Information 9 Bit score represents the information content of five conserved domains mapped using the MEME tool (A) Position and multiple sequence comparisons of the five conserved structural domains among CmOMTs, with different colors indicating

10.7717/peerj.16483/supp-10 Supplemental Information 10 Predicted 3-D structures of CmOMT proteins

10.7717/peerj.16483/supp-11 Supplemental Information 11 Schematic diagram of the synthesis of *CmOMTs* (A) Interchromosomal relationships between *CmOMTs* in melons. Synteny analysis of *OMTs* between melon and *Arabidopsis* (B), watermelon (C), and cucumber (D). Gray lines indicate collinear gene pairs and red lines represent collinear *OMT* gene pairs. *C. melo*, *A. thaliana*, *C. lanatus*, and *C. sativus* represent melon, *Arabidopsis*, watermelon, and cucumbers, respectively.

10.7717/peerj.16483/supp-12 Supplemental Information 12 Correlation analysis between *CmOMTs* and resistance genes

10.7717/peerj.16483/supp-13 Supplemental Information 13 Raw data of qPCR

## Additional Information and Declarations

### Data Availability

The following information was supplied regarding data availability: The raw measurements are available in the Supplementary File.
