# Supplementary figures and images for: Genome-wide identification of the OMT gene family in Cucumis melo L. and expression analysis under abiotic and biotic stress
Source: PeerJ. 2023 Dec 14;11:e16483. doi: 10.7717/peerj.16483 (PMC10725674; doi:10.7717/peerj.16483)

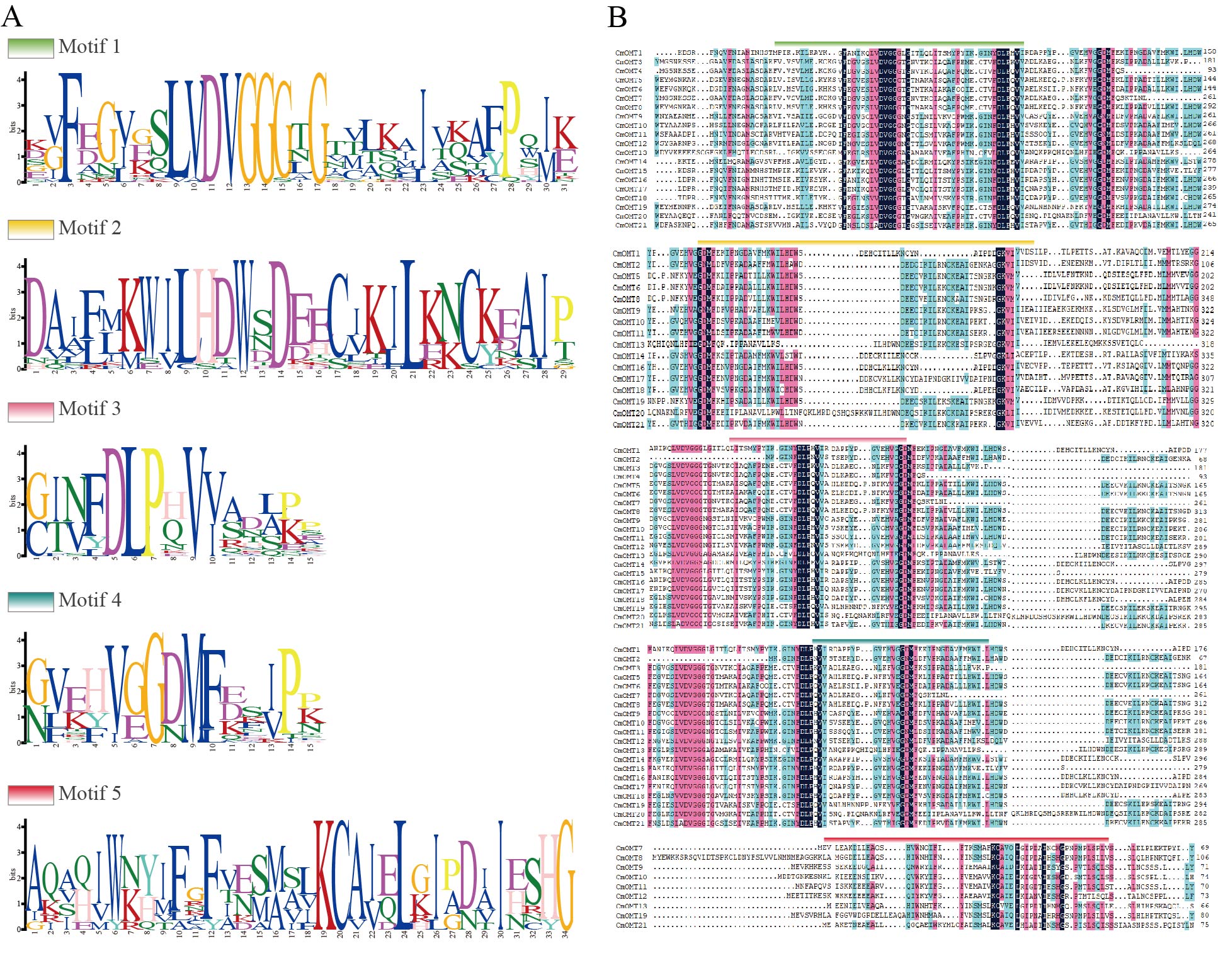

Supplement: Supplemental Information 9 — Position and multiple sequence comparisons of the five conserved structural domains among CmOMTs, with different colors indicating [file peerj-11-16483-s009.jpg]

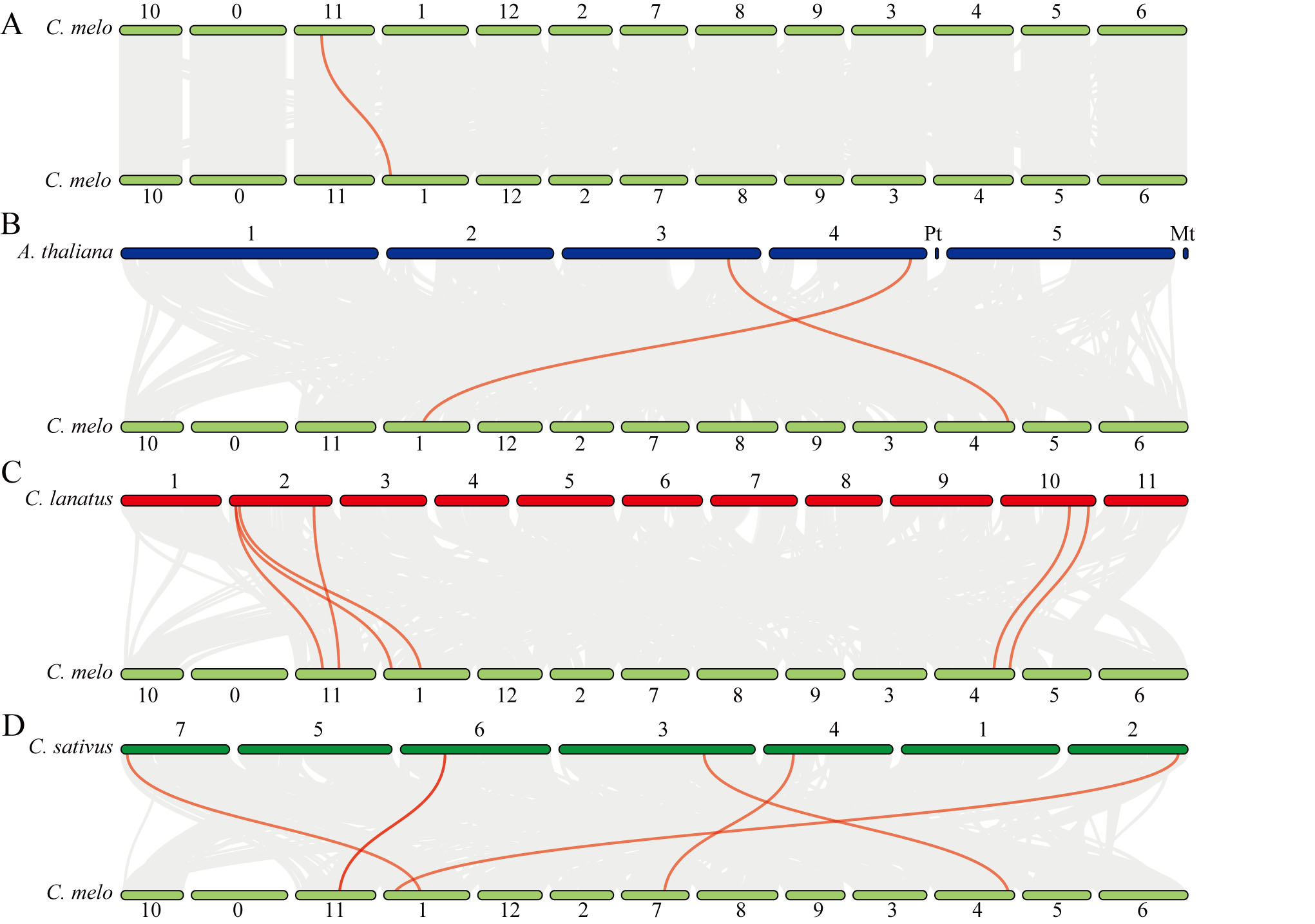

Supplement: Supplemental Information 11 — (A) Interchromosomal relationships between CmOMTs in melons. Synteny analysis of OMTs between melon and Arabidopsis (B), watermelon (C), and cucumber (D). Gray lines indicate collinear gene pairs and red lines represent collinear OMT gene pairs. C. melo, A. thaliana, C. lanatus, and C. sativus represent melon, Arabidopsis, watermelon, and cucumbers, respectively. [file peerj-11-16483-s011.jpg]

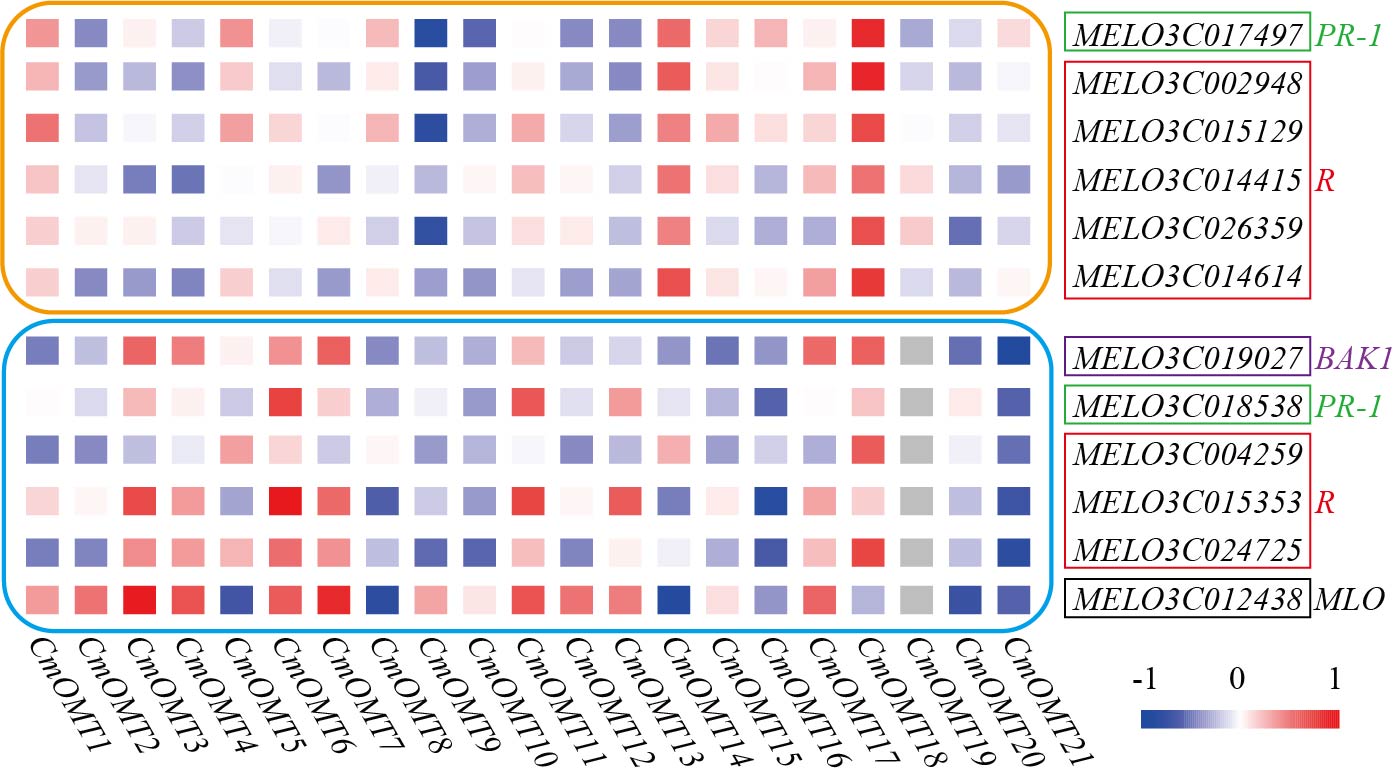

Supplement: Supplemental Information 12 [file peerj-11-16483-s012.jpg]
