# Supplementary material for: Genome-wide identification of the OMT gene family in Cucumis melo L. and expression analysis under abiotic and biotic stress
Source: PeerJ. 2023 Dec 14;11:e16483. doi: 10.7717/peerj.16483 (PMC10725674; doi:10.7717/peerj.16483)
Supplement: Supplemental Information 10 [file peerj-11-16483-s010.pdf]

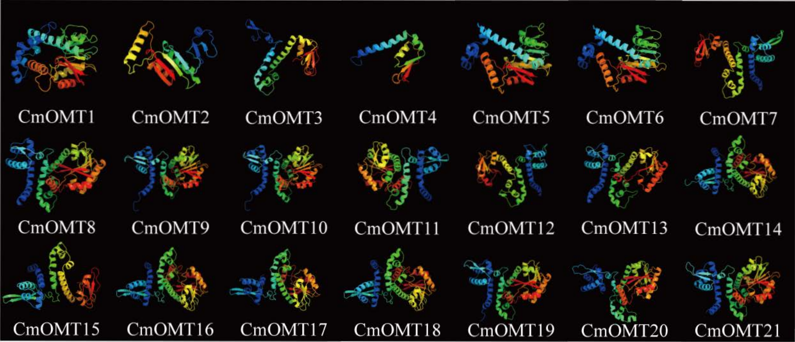
CmOMT1
CmOMT2
CmOMT3
CmOMT4
CmOMT5
CmOMT6
CmOMT7
CmOMT8
CmOMT9
CmOMT10
CmOMT11
CmOMT12
CmOMT13
CmOMT14
CmOMT15
CmOMT16
CmOMT17
CmOMT18
CmOMT19
CmOMT20
CmOMT21
